# Progress of estrogen receptor and spliceosome in endometrial carcinoma

**DOI:** 10.3389/fendo.2025.1586191

**Published:** 2025-08-25

**Authors:** Ziqi Hong, Ting Mo, Peiquan Zhou, Jian Chen, Xin Li

**Affiliations:** ^1^ Guangxi Key Laboratory of Tumor Immunology and Microenvironmental Regulation, Guilin Medical University, Guilin, China; ^2^ Department of Neurosurgery, Affiliated Hospital of Guilin Medical University, Guilin, China

**Keywords:** estrogen, estrogen receptor, isoform, endometrial cancer, molecular mechanism

## Abstract

Endometrial cancer (EC) is one of the most common gynecological cancers in developed countries. Like EC, most female reproductive tract malignancies are thought to be hormonally driven, with estrogen signaling acting as an oncogenic signal. The actions of estrogen are mediated through the classical nuclear estrogen receptors α (ER-α) and β (ER-β) as well as transmembrane G protein-coupled estrogen receptors (GPR30 and GPER). Ligand-bound estrogen receptor (ER) and GPER trigger multiple downstream signaling pathways that regulate the cell cycle, differentiation, migration, and apoptosis in various tissues, including the endometrium. Additionally, growing evidence suggests that selective splicing events at the receptor result in multiple ERα proteins with different molecular weights and functional structural domains. Examples include ER-α66, ER-α46, and ER-α36. In addition, various post-translational modifications (PTMs) further affect ER-α cellular localization and ligand affinity, resulting in a change in the cellular function. These splice isoforms and PTMs are differentially expressed in a tissue-specific manner. They mediate some aspects of ER-α signaling and may even antagonize full-length ER-α. Therefore, both ER-α and its splice isoforms may play a role in the development of EC. In this review, we examine the influential roles of ER-α and ER-β, as well as the GPER estrogen signaling pathway, in EC. Our goal is to provide theoretical support for further research on the molecular mechanisms between ER and EC and to generate new ideas for the early diagnosis of EC and the development of new drugs.

## 1 Introduction

### 1.1 Background and epidemiology of endometrial cancer

Endometrial cancer (EC) is a malignant tumor that originates in the glandular epithelium of the endometrium. It is the most common cancer of the female reproductive tract. The population is growing rapidly worldwide, especially in Europe and the United States (1). Not only the occurrence of EC associated with excessive estrogen secretion but also its major risk factor is the prolonged and unbalanced exposure of the endometrial glandular epithelium to estrogen. Based on histopathologic features, there are two types of EC: type 1 and type 2. Type 1 is endometrioid and estrogen-responsive and accounts for 80%–85% of all EC (2). In addition, type I are primarily driven by hormones and are considered low-grade carcinomas with glandular structures that usually express high levels of ER-α (3). In recent years, the morbidity and mortality rates of EC patients have continued to rise worldwide, despite increasingly advanced diagnostic techniques and therapeutic treatments. The biological activity and therapeutic response of this disease are affected by multiple histologic and molecular-level abnormalities (4). Therefore, to accurately assess patient risk and select the most appropriate treatment, researchers consider the patient’s age, family history, histologic typing and grading, and the local and systemic progression of the disease (5, 6).

### 1.2 Background introduction of estrogen receptor

It is well known that estrogen exerts a critical influence on the female reproductive system through two distinct nuclear receptor (NR) isoforms, ER-α and ER-β (ERs), and probably via GPER. ERs belong to the NR superfamily and are determined by the ESR1 and ESR2 genes. In the classical model of estrogen action, ligand-activated transcription factors bind directly to specific estrogen-responsive elements (EREs) in the DNA in order to regulate transcription of their target genes (7). In addition to ERs, the GPER, a seven times transmembrane receptor, mediates estrogenic effects not as a transcription factor binding to the ERE but through non-genomic signaling. Further investigation is necessary to determine the expression levels and molecular mechanisms of these three receptors in normal and cancerous tissues.

#### 1.2.1 Estrogen signaling in normal endometrium

It is reported that ERs are expressed in the murine uterine (8). ER-α can act on endometrial cells via estrogen signaling to promote their proliferation, which can ultimately contribute to EC. Furthermore, some studies suggest that the incorrect biological effects of ERs can cause diseases such as EC (9). However, ER-β has an opposite function to ER-α. While several *in-vitro* studies have suggested that ER-β acts as a growth inhibitor in EC cell lines, the results of ER-β expression in EC contradict this. Moreover, the latest research indicates that non-specific antibodies are used for all types of ER-β expression in various immunohistochemistry (IHC) studies. Additionally, ER-β may act as a tumor suppressor in healthy or minimally dedifferentiated endometrial tissues; however, it functions as a tumor promoter in fully dedifferentiated, high-grade EC (10). In short, the research findings indicate that the expression and signaling of ER-β are linked to the pathophysiology of tumors (11–13). ER-β can sometimes act as an antagonist of ER-α (14). GPER, formerly known as GPR30, has multiple mechanisms of action; on the one hand, it mobilizes calcium and initiates cyclic adinosine monophosphate(cAMP) synthesis, and on the other hand, it trans-activates the epidermal growth factor receptor (EGFR) and induced mechanisms such as PI3K and MAPK signaling pathways. These mechanisms ultimately lead to GPER signaling, which affects gene regulation, cell cycle progression, cell proliferation, differentiation, apoptosis, migration, and invasion. This makes GPER signaling an important player in carcinogenesis (15).

#### 1.2.2 Dysregulation in EC

ER-α plays a major role in EC, with higher expression levels than ER-β. Conversely, ER-β is responsible for maintaining cell renewal and regeneration in normal endometrial tissue. Changes in the ratio between the two affect the occurrence of EC. Additionally, ER-α expression is high in the early stages of EC and gradually decreases in the late stages. Studies have shown that decreased ER-α expression in EC is associated with tumor differentiation grade and lymph node involvement but not significantly related to incidence or overall survival. High ERβ expression has been associated with shorter disease-free survival in patients with EC and regional lymph node metastasis (16). By examining ERs and GPER mRNA and protein levels, as well as their intracellular protein distribution in EC tissue and adjacent control endometrial tissue, the investigators found that immunoreactivity for ERα was weaker in EC tissue than in adjacent endometrial tissue. IHC showed light but unaltered cytosolic nuclear staining, and mRNA and protein levels showed decreased ER-α expression in EC tissues. For ER-β, nuclear and cytoplasmic immunoreactivity remained unchanged in both tissue types; however, EC tissue exhibited lower mRNA and protein levels compared to adjacent endometrial tissue (16). While GPER mRNA levels remained unchanged, a notable decrease was observed in its protein expression levels. The Hojnik research team evaluated the co-expression levels of ER-α, ER-β, and GPER in EC tissue and compared them with normal endometrial tissue as a control. They used Spearman’s correlation coefficient to calculate the rank correlation between the mRNA and protein expression levels of related genes and the IHC score expression levels. The results revealed a statistically significant correlation among ESR1, ESR2, and GPER expression, yet no correlation between GPER and ESR1 or ESR2 expression.

## 2 Biological basis of estrogen receptors

### 2.1 Structure of the estrogen receptors

**Figure 1:**
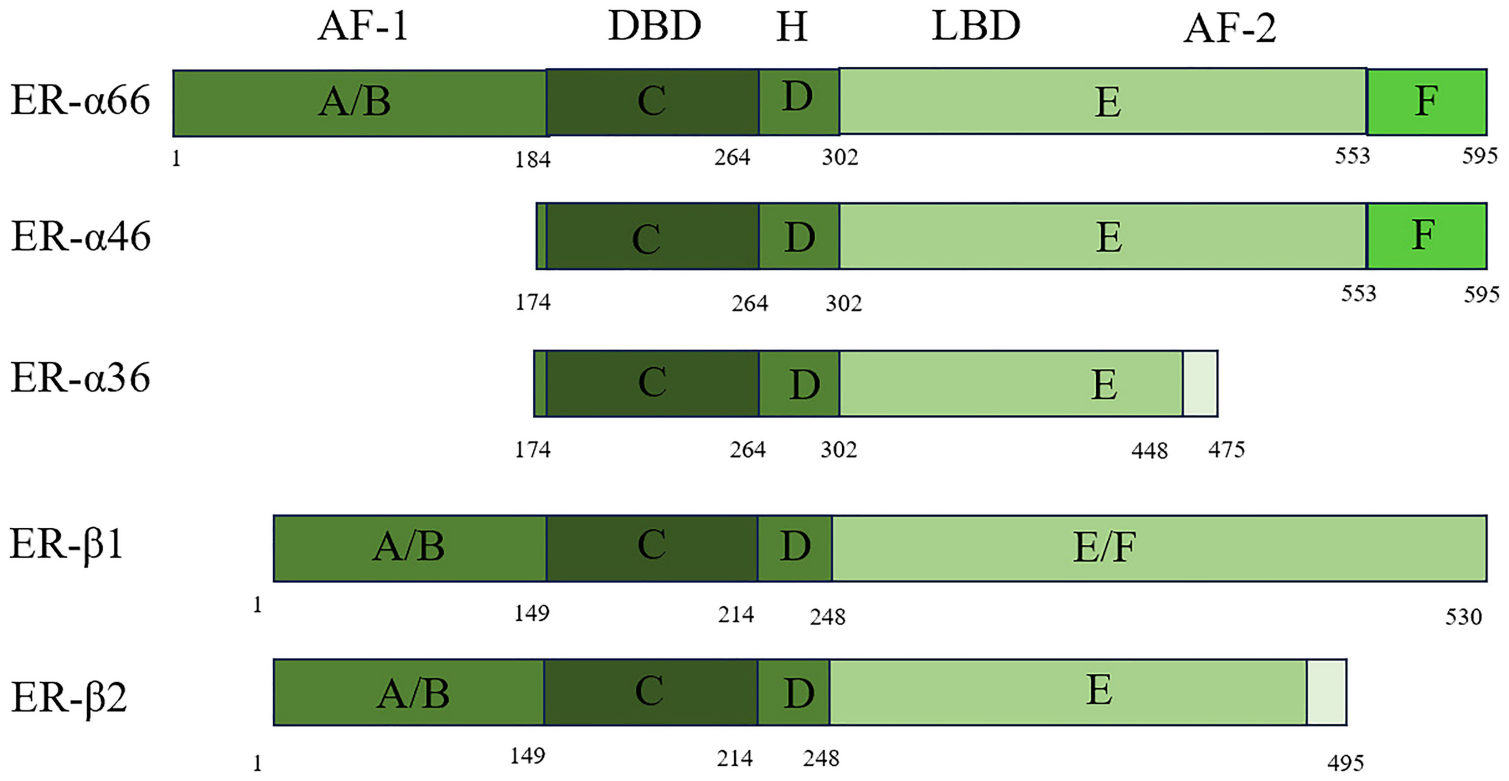
Scheme of ER-α66, ER-α46, ER-α36, ER-β1, and ER-β2 structure.

The ER/ER-α66 is a member of the NR superfamily, and NRs are categorized as representing a variety of ligand-dependent transcription factors that have a conserved functional domain structure. As a transcriptional regulator, NR contains a DNA-binding domain (DBD) and a transcriptional activation domain (17). It consists of three parts: the A/B region, which binds to the amino terminus; the central C region, which binds to the DNA; and the D/E/F region, which binds to the ligand. These three components are independent of each other and interact. When the amino-terminal structures of the A/B domains are encountered, the ERs, which share 17% amino acid identity, act as ligand-independent activation function 1 (AF-1) and are involved in inter- and intramolecular interactions and activation of gene transcription (18). The central C region, which is nearly identical to the amino acid sequence, is the DBD that enables ER dimerization. The D domain contains the nuclear localization signal and has 36% amino acid identity. This domain is important for receptor dimerization and binding to chaperone heat shock proteins. It also connects the central C region to the multifunctional C-terminal (E) domain. The E domain is a ligand-binding domain (LBD) with 56% amino acid identity between ERs. It is a globular region in which the LBD works with the amino-terminal domain to regulate gene transcription. The F domain is located at the extreme carboxyl terminus of the receptor and has 18% amino acid identity with ERs (19, 20). The F domain of ER-α is involved in regulating transcriptional activity, coactivator interactions, dimerization, and stability. This region is generally known as the AF-2 structural domain (21). Splicing variants are a common and flexible mechanism of genetic expression in mammalian cells. To date, three variants have been discovered: ER-α66, ER-α46, and ER-α36. Their specific structures and differences are shown in **Figure 1** below.

ER-β belongs to the steroid hormone receptor family, just like ER-α. ER-β exists in two forms: ER-β1 and ER-β2 (**Figure 1**). Despite sharing only moderate homology (58% in humans) at the LBD, ER-β1 binds to ER-β2 with high affinity through its LBD. Although they share only moderate homology at the protein level (58% in humans) at the LBD ER-β1 binds to ER-β2 with high affinity through its LBD. ER-β and ER-α have almost identical DBDs, but in contrast to their LBDs, the homology is only 60% (22).

### 2.2 Biological functions of estrogen receptors

The ovaries produce hormones such as testosterone, luteinizing hormone(LH), and estrogen, which play a vital role not only in the ovaries but also in the endometrium (23). Unlike other mucosal epithelia, the endometrium responds significantly to estrogen and progesterone. Estrogen and progesterone regulate the cyclical proliferation and differentiation of the endometrial glands. This is accompanied by the temporal and spatial expression of their receptors ER-α and the progesterone receptor (PR) (24, 25). Estrogen is an essential sex steroid hormone that regulates physiological and pathophysiological processes and plays a vital role in human life (26). In the normal endometrium, estrogen acts as a mitogen and drives tissue growth during the menstrual cycle as part of pregnancy anticipation (27). Estrogens regulate the expression of their target genes by binding to their cognate receptors, ER-α and ER-β (28). The menstrual cycle is divided into four phases based on hormonal changes: menstruation, the follicular phase, ovulation, and the luteal phase. During the follicular phase of the menstrual cycle, the developing follicle produces estrogens. The most prominent of these are estrone (E1) and 17β-estradiol (E2), which work together to promote endometrial growth. Estrogen production peaks at ovulation, which occurs at the end of the follicular phase. However, estrogen is produced by the corpus luteum during the mid- and late luteal phases, and its production declines before menstruation (29). LH is a steroid hormone produced by the placenta, ovaries, and adrenal glands. It is essential for reproductive function and the regulation of the menstrual cycle. LH initiates and maintains ovulation and pregnancy. It also has a significant protective effect on a woman’s uterine lining. The second estrogen secretion does not lead to endometrial cell proliferation precisely because progesterone is present. During the follicular phase of the menstrual cycle, LH levels are low. They rise during the mid- to late luteal phase due to the formation of the corpus luteum. Progesterone inhibits estrogen-induced endometrial growth during the luteal phase. When progesterone production is inadequate, the risk of EC significantly increases (30). The body has a natural balance between estrogen, which promotes growth, and progesterone, which is resistant to growth. However, in the development of cancer, estrogen typically has a stronger influence. In animal models, high levels of estrogen resulting from insufficient progesterone activity can trigger endometrial hyperplasia or cancer. This suggests that an estrogen/progesterone imbalance can lead to EC in its early stages (31, 32). In summary, it is well-established that estrogen plays a role in suppressing EC initiation and development.

### 2.3 Selective splicing events of the estrogen receptor

Abnormal splicing of proteins is a very common mechanism in cancer. Neoplastic tumors use this process to express genes that promote their growth and survival. Variable splicing events of pre-messenger RNAs are ubiquitous in mammals and regulate gene expression in a flexible manner (33). A single gene locus can give cells an opportunity to create different types of protein isoforms that have opposite functions. The spliceosome, a protein complex consisting of five small ribonucleoproteins, is directly involved in selective splicing events (34, 35). These molecules communicate with a number of transacting factors that recognize cis-regulatory sequences within pre-mRNAs. These factors then direct the generation of splice variants by different mechanisms, including the preferential use of alternative promoters, exons, or splice sites, as well as alternative sites for polyadenylation. Not only are variable splicing events based on providing proteomic diversity to provide a significant evolutionary advantage, but they are also beneficial to remodel protein–protein interaction networks typically regulated in a tissue-specific manner. The loss of splicing event fidelity is ubiquitous in cancer cells. There are generally two ways that cancer cells gain an early growth advantage. One way is through reorganization of the splicing profile. The other way is through conversion to cells that express specific splice isoforms. Therefore, specific splicing errors can be detected in developing cancer cells compared to normal-looking tissues in pathological situations. Genome-wide studies have confirmed cancer-specific splice variant events. In the early stages of cancer, regulating selective splicing events benefits nascent cancer cells if the proteins encoded by spliceosome isoforms stimulate cell proliferation and inhibit apoptosis. This drives the growth of uncontrolled cells. The ER is the primary mediator of estrogen signaling. Evidence has revealed that the regulation of ER-α gene transcription, splicing, and expression across tissues is intricate (36). In addition, there are multiple ER-α proteins with different molecular weights and functional domains, like, there is a variety of PTMs. These behaviors further alter the cellular localization of ER-α as well as ligand affinity, thereby affecting cellular function (37). These isoforms and PTMs are differentially expressed in a tissue-specific manner, mediate specific aspects of ER-α signaling, and may even act antagonistically to full-length ER-α. In addition, aberrant variable splicing events of proteins have been mostly reported in human cancers. ER-α36 (38), as a splice variant of the ER1 locus, not only can control estrogen-triggered non-genomic membrane signaling pathways but also has the anti-4-hydroxytamoxifen activity in breast cancer therapy. This certainly gives us new ideas in breast cancer treatment and in estrogen-related tumors. Although little is known about the expression and function of ER-α splicing variants in EC, we will include a discussion of the study results when they are available. Our current understanding of their properties and physiological functions is summarized in **Table 1**.

**Table 1:** This table summarizes the molecular weight, size, location, structural-functional features, and experimental findings of the five spliceosomes of ERα (ER-α46, ER-α36, ER-α66, ER-α-LBD, ER-αV/ER-αΔ3-6.

Isoform	MW (kDa)	Location	Structural features	Experimental exploration
ER-α46	46	On the cell membrane of uterine natural killer (uNK) cells or peripheral blood leukocytes	lacking the first 173 N-terminal amino acids sharing an identical DNA sequence with ER-α66 for the remaining portion	was first found in MCF-7 mutant human breast cancer cell lines and named ER-α46 suggesting that ER-α46 may be involved in membrane-initiated estrogenic signaling powerfully inhibits the AF-1 activity of ER-α66
ER-α36	36	Located on the plasma membrane of glandular cells in both EC tissues and cell lines	lacking the transcriptional activation domain of AF-1 and AF-2 and has a unique addition of 27 amino acids on the C terminus encoded by a previously unknown exon	The result of ER-α36 expression in human EC tissues revealed that ER-α36 was implicated in invasion and differentiation of EC Its expression has been reported at least in normal endometrial tissues with significantly higher levels than endometrial carcinoma tissues
ER-α66	66	Localized in the nucleus and involved in estrogen signaling	Including a DNA binding domain (DBD) and ligand binding domain (LBD) As a ligand for activation or repression of ER-mediated transcriptional activity derived from two transactivation domains, AF-1 and AF-2	Compared to control endometrial tissue, immunoreactivity for ER-α in EC tissue was weaker for nuclei with minor, but unchanged, cytoplasmic staining; mRNA and protein levels showed decreased patterns for ER-α in EC tissue
ER-α-LBD	37.3	It was largely found in the cytoplasm and mitochondria of breast cancer cell lines	Laking the N-terminal domains including AF-1, DBD, and a part of hinge region, but be composed of LBD and AF-2	Overexpression of ER-αLBD promoted breast cancer growth and made cancer cells resistant to ER-α antagonist treatment It has been reported in human uterine endometrial tissue from uterine leiomyoma patients
ER-αV/ER-αΔ3-6	37	Acting on the protein E3-3 (NPE3-3) and binds to ER-α	Lacking the large portions of DNA- and ligand binding domains that are encoded by exons 3-6	ER-αV variant expressed in normal tissues

## 3 Estrogen receptors remodeling of TME in EC

ERs and GPER/GPR30 are key mediators of estrogen function. The first estrogen-related receptor.ER-α, the first estrogen-related receptor, was discovered in 1962. Until 1996, it was believed that estrogen action could only be activated by this receptor (39). However, when another highly homologous ER, ER-β, was discovered, this belief changed. Then, as the research progressed, a third ER, GPER/GPR30, was introduced in 2000 (40, 41). ER function can be caught by many therapeutic approaches for estrogen-related diseases. It is important to understand the mechanisms of these receptors to optimize these techniques. Several studies have been conducted on the expression and role of ER-β in EC with conflicting results, but analysis of TCGA data shows that the average expression level of ER-α (2.9-fold) is much higher than ER-β in endometrial tumors (42).

### 3.1 Mechanisms of ER-α-mediated signaling

ER-α acts as a transcription factor involved in regulating gene expression during the cell cycle, proliferation, and apoptosis. After ER-α binds to E2, the receptor changes its conformation and undergoes dimerization and translocation to the nucleus, where it interacts with transcriptional coactivators in an active form (43). However, antagonists such as tamoxifen induce an inactive ER-α conformation, enabling ER-α to recruit co-repressor proteins. This process enables ligand-activated ER-α to bind to EREs within the promoters of target genes. ERα can also interact with transcription factors such as activator protein 1 (AP-1) and specificity protein 1 (SP-1) via serum response elements (SREs) to regulate genes lacking EREs in their promoters (44). This genomic action accordingly regulates the transcription of hundreds of target genes involved in cell growth and differentiation. Biological responses to ER-α, as well as the dysregulation of co-regulators and target proteins, play an important role in the development of most EC. In general, ERα activation has been associated with the production of second messengers, such as cAMP, and involves the stimulation of the PI3K/AKT or Ras/MAPK signaling pathways. MAPK3, alternatively referred to as ERK, constitutes a constituent of the MAPK family. MAPKs are involved in a variety of cellular functions, including mitosis, metabolism, survival, cell death, differentiation, and gene expression. MAPKs can also regulate estrogen signaling. Previous studies have demonstrated that the activation of the MAPK pathway is associated with hormone-driven progression of malignant tumors, such as EC and breast cancer (45).

The MAPK/ERK signaling pathway influences the chemoresistance of breast cancer cells to aztreonamycin and paclitaxel (46). This suggests that aberrant activation of the pathway may be a key factor in tumor treatment resistance. However, research on the role of the MAPK/ERK pathway in EC, particularly in regulating estrogen metabolism, remains limited. Because EC is a hormone-dependent tumor, it is important to explore the mechanism by which estrogen and its receptors (e.g., ER and GPER) promote proliferation through the MAPK/ERK pathway. Qing Wang’s (47) team used Chinese medicine physiotherapy as a starting point to validate the role of resveratrol (Res), a drug structurally similar to estrogen, in regulating the MAPK/ERK pathway. The study found that Res can regulate estrogen metabolism by activating the MAPK/ERK pathway. First, an experimental approach was undertaken to ascertain whether ERK is a target of Res. This entailed examining the phosphorylation status of ERK following exposure to Res. The results demonstrated that pretreatment with Res led to an augmentation in phosphorylated ERK levels. Conversely, pretreatment with ERKi resulted in a suppression of ERK activation by Res. These findings imply that MAPK/ERK activation may be multifactorial in nature. Res may activate downstream signaling pathways, such as MAPK/ERK, by binding to ERs, such as ERα and ERβ, or to GPER through direct or indirect action. Aberrant activation of the MAPK/ERK pathway is closely associated with EC cell proliferation and chemoresistance. This suggests that Res may influence EC progression or treatment by modulating this pathway. Further research is needed to determine whether the MAPK/ERK pathway simultaneously affects EC chemotherapy sensitivity and hormone-driven proliferation.

### 3.2 Mechanisms of ERβ-mediated signaling

The effects of ER-β, namely its capacity to inhibit cell proliferation and induce apoptosis, are contingent upon factors such as the specific tissue under study, the prevailing cellular environment, the presence of transcriptional co-activators, and the expression of ER-α. Despite the paucity of discoveries concerning ER-β in EC insights from breast cancer research can offer valuable guidance. In the context of breast cancer, the expression of ER-β in tumor cells is often associated with a more positive prognosis for patients. A body of research has demonstrated that ER-β and its isoforms, in conjunction with co-regulators such as AIB1, NF-κB, and TIF-2, have a tendency to co-regulate the proliferation and progression of breast cancer cells (48). These studies indicate that ER-β plays a role in mediating proliferation in breast cancer. Consequently, further experimentation is necessary to ascertain whether this receptor exerts a comparable effect in EC. Additionally, the expression of different splicing variants of ER-β mRNA leads to the translation of functionally distinct receptor proteins, thereby increasing the complexity of ER-β signaling and its role in the endometrium. Chakravarty’s group studied 57 samples of proliferative endometrium versus 26 samples of endometrial carcinoma, based on RT-PCR, and showed reduced expression of ER-β in endometrioid carcinomas compared with proliferative endometrium (49). In contrast, studies by several research groups have found no changes in ER-β1 and ER-β2 expression in EC compared to postmenopausal endometrium (50). These observations suggest the potential for these splice variants to exhibit a close relationship with hormone levels *in vivo*, warranting further investigation. In addition, Häring’s group categorized the group of patients with endometrial-like EC, who underwent radical resection, with an age range of 54–82 years, into three graded subgroups (G1, 15 patients; G2, 16 patients; and 15 patients with G3 type). Using RT-qPCR, they analyzed 28 normal endometrial tissues and 46 EC tissues and examined the expression of 18 ER-β splice variants. Their results showed that ER-β5 and three exon-deletion ERβ variants were overexpressed in EC. Previous studies have reported ER-β5 overexpression in EC, as well as in breast and ovarian cancers (51, 52). These findings suggest that ER-β5 plays a role in promoting tumorigenesis and development in EC, which is supported by the fact that ER-β5 and two other ER-β variants are particularly highly expressed in G3 tumors compared to G1 or G2 tumors and postmenopausal endometrium (53). Based on this, the Collins group also proposed an oncogenic role for ER-β5 in EC. They observed that ER-β5 formed heterodimers with ER-α in Ishikawa EC cells, which increased their sensitivity to E2. The authors hypothesized that ER-β5 expression in endometrial epithelial cells may increase the risk of malignant transformation. This confirms the tumor growth-promoting role of ER-β5 in EC (54). In addition, Häring’s group identified a unique ER-β splice variant with exon 4 deletion, ER-βΔ4, which was significantly downregulated in G2 and G3 tumors as well as in total EC samples. Conversely, we found that seven ER-β splice variants (including ER-β1 and ER-β2) were positively correlated with HER2 expression in EC tissues (53). This could indicate that most ER-β splice variants are involved in the development of EC.

### 3.3 Mechanisms of GPER1-mediated signaling

The rapid action of E2 in the development of EC is also majorly mediated by GPER, which is a cellular mediator. It can activate metalloproteinases and induce the release of heparin-binding epidermal growth factor, which binds to and activates the EGFR (55). A number of studies have examined the biological interactions between growth factors, such as EGFR or IGFR, and sex steroid hormones, including estrogens and androgens (56, 57). This interaction leads to the activation of downstream signal transduction molecules, including the production of cAMP or the activation of growth factor receptors, the PI3K/AKT pathway, or the Ras/MAPK pathway. GPER is stimulated by E2 to increase cAMP concentration and mobilize intracellular calcium (58). This activates signal transduction cascades, such as PI3K/AKT and Ras/MAPK, which ultimately regulate the transcription of genes involved in the proliferation and survival of EC cells. These genes include c-fos and cyclin D, B, and A, which participate in the cell cycle and promote proliferation. GPER induces them, while it downregulates other genes involved in the apoptosis process, such as BAX, caspase 3, and BCL2 (59).

In a thymus-free mouse xenograft model of EC, GPER activation promoted RL95–2 cell of EC formation. Another study found that blocking GPER halted tumor growth in an athymic mouse model with an HEC-1A EC xenograft. The findings of these studies have demonstrated that growth factors (e.g., EGFR or IGFR) interact with sex steroid hormones (e.g., estrogens and androgens) through receptors such as GPER (60). This interaction activates signaling pathways, such as cAMP, PI3K/AKT, and Ras/MAPK, and regulates genes related to cell proliferation (e.g., c-fos, cyclins) and apoptosis (e.g., BAX, caspase-3). This activation promotes EC cell proliferation. In contrast, GPER blockade inhibits EC cell proliferation.

As mentioned above, the activation and blockade of GPER play distinct roles in animal xenograft models of EC. Additionally, we learned that high GPER expression is present in at least four types of human cancers, including thyroid, breast, ovarian, and EC. These results suggest that GPER is a promising biomarker for EC progression. Several *in-vitro* studies have shown that the specific GPER agonist G-1 can inhibit tumor growth. In a recent study, the agonist G-1 demonstrated different GPER expression levels in EC cell lines (RL-95-2, HEC-1A, and HEC-1B) and could obviously suppress cell growth in a dose-dependent manner (11). However, studies on negative HEC-1B GPER have found no effects. Several studies have revealed that the agonists G-1 and E2 have been reported to be carcinogenic for EC cell lines. In a study by Du et al., the expression levels of GPER protein and mRNA in Ishikawa and KLE EC cell lines were up-regulated by G-1 and E2. Furthermore, GPER knockdown and interruption of the MAPK signaling pathway nearly completely inhibited the proliferation induced by G-1 and E2 (61, 62). Consequently, it is concluded that GPER is ineffective in negative cells. In contrast, G-1 and E2 promote EC cell proliferation by activating the MAPK pathway through upregulation of GPER. Furthermore, the results demonstrate that knockdown of GPER or blockade of MAPK inhibits this effect. The above findings suggest that GPER acts in a cell-dependent manner. Its activation in GPER-positive cells may promote or suppress EC cells through pathways such as MAPK, whereas it has no significant effect in GPER-negative cells. This phenomenon is paradoxical and suggests that the regulatory mechanism of GPER is complex, possibly involving pathway crosstalk or cell-specific signaling networks.

### 3.4 Signal transduction and endoplasmic reticulum gene action

The endoplasmic reticulum binds to the distal regulatory region of the promoter. In estrogen signaling, the ER-binding region is at least 5 kb away from the promoter. Such binding accounts for approximately 95% of the total (63). Indeed, although the endoplasmic reticulum interacts with distal promoters, most estrogen-induced transcription is driven by gene expression enhancers. Through long-range loop interactions, these enhancers communicate with the target gene of the promoter. According to CHIA-PET data, the ER is directly involved in these loop interactions, with approximately 10% of the ER binding to the site. Evidence suggests that when the ER binds, it loops to another region. Consistent with this observation, ER binding sites are approximately 10-fold more abundant than genes whose expression is altered in response to E2 treatment in endometrial and breast cancer cells.

### 3.5 Interaction with other steroid hormone receptors

Steroid hormones and their receptors in the endometrium exhibit dynamic expression patterns that drive the cycle of growth, shedding, and regeneration of the endometrium. These receptors can bind to similar sequences and influence each other’s actions. Vahrenkamp et al. analyzed the association between steroid hormone receptor expression at the mRNA level and outcomes for EC patients based on TCGA data. While nearly all type I endometrial tumors express ER, higher expression is associated with a better prognosis. This association may be due to the fact that high ER expression indicates a higher degree of hormone-driven tumor differentiation. Not surprisingly, high PR expression was also associated with a better prognosis. ER and PR expression in endometrial tumors are highly correlated, and ER directly regulates PR expression (64).

## 4 Estrogen receptor as a target for EC therapy

Researchers have consistently sought to develop and strengthen effective treatments for EC. Studies on the molecular mechanisms of breast cancer have established a solid foundation for our understanding of estrogen signaling in carcinogenesis. However, despite similar phenotypic consequences of estrogen signaling, there are many differences in ERs between endometrial and breast cancers. Therefore, the role of ER signaling in EC remains to be determined. As described above, we clearly found that, compared with breast cancer cells, ER genomic binding in EC cells is controlled by different and yet unknown transcription factors (65–67). In addition, EC appears to have different cofactors than breast cancer. Thus, we will not only study EC-specific transcription factors and cofactors but also elucidate the way in which cell type-specific gene regulation occurs through the endoplasmic reticulum. This will help us discover some interesting biological phenomena. Discovering EC-specific ER-influencing transcription factors and ER-binding cofactors may reveal clinical vulnerabilities that could be exploited to treat EC (68, 69).

## 5 Conclusion

In summary, this review provides an overview of the structural diversity and biological significance of ER isoforms and their implications for tumor microenvironment remodeling. Current research shows that continuous exposure to estrogen increases cancer risk, especially emphasizing the mechanistic relevance of ER-related splicing events and PTMs in shaping estrogen signaling in EC. We also discussed the potential of ER as a therapeutic target for EC.

An increasing number of discoveries have suggested that some novel factors and molecular targets are significant in prognostication. For example, Enrico et al. observed that a statistical analysis of low-risk EC patients revealed a higher recurrence rate of L1CAM in the distal region. Therefore, it demonstrated that the L1CAM can be a predictor of poor outcome in endometrial carcinoma. Additionally, Andrea et al. conducted a systematic review and meta-analysis of the relationship between L1CAM expression and survival rates in type I endometrial carcinoma (EC), confirming that L1CAM expression is associated with EC invasive behavior. This protein can be easily detected through preoperative biopsy. There are already clinical applications of the same glycoprotein as an antibody-mediated therapeutic target (70, 71).

As mentioned earlier, the potential of ER as a therapeutic target for EC suggests that small molecule biomarkers may represent a breakthrough in disease treatment. The functional role of ER in EC is becoming clearer day by day. ER, as a transcription factor, is a carcinogenic signal in EC, which promotes the transcription of downstream target protein through the ERE signaling pathway. Therefore, it can prompt the occurrence and development of EC with the induction of proliferation, invasion, metastasis, and anti-apoptosis. Although some research progress discovery has been made regarding ER-α in targeted therapy of breast cancer, anti-ER-α therapy in EC still needs to be further explored in depth. Further exploration of the biological behavior of EC and its molecular mechanisms, based on continuous and profound research on ER subtypes, will help us uncover early diagnosis of EC as well as potential therapeutic targets.
